# 8p23.1 duplication syndrome: narrowing of critical interval to 1.80 Mbp

**DOI:** 10.1186/s13039-014-0094-3

**Published:** 2014-12-09

**Authors:** Axel Weber, Angelika Köhler, Andreas Hahn, Ulrich Müller

**Affiliations:** Institut für Humangenetik, Justus-Liebig-Universität, Schlangenzahl 14, 35392 Giessen, Germany; Klinik für Kinderneurologie und Sozialpädiatrie, Justus-Liebig-Universität, Giessen, Germany

**Keywords:** 8p23.1 duplication syndrome, 8p23.1, SOX7, TNKS1, Developmental delay, Intellectual disability, SNP array

## Abstract

**Background:**

A 3.68 Mbp duplication of 8p23.1 defines the 8p23.1 duplication syndrome. The main features of this syndrome are developmental delay and/or learning problems.

**Results:**

Here we present a patient with a 1.80 Mbp duplication in 8p23.1 and characteristic signs and symptoms of the syndrome, including delay of motor and speech development and intellectual disability.

**Discussion:**

The case indicates that genes within this interval, in particular dosage sensitive genes *SOX7* and *TNKS1,* and possibly *MIR124-1* and *MIR598* as well suffice to cause the pathognomonic features of the 8p23.1 duplication syndrome.

**Electronic supplementary material:**

The online version of this article (doi:10.1186/s13039-014-0094-3) contains supplementary material, which is available to authorized users.

## Background

Duplication of a core region of 3.68 Mbp in 8p23.1 can be considered a pathogenic copy number variant (pCNV) and accounts for the clinical findings in the 8p23.1 duplication syndrome [1]. This syndrome is characterized by developmental delay and/or learning problems in most cases (>90%). Behavioral abnormalities such as attention deficit hyperactivity disorder (ADHD) have been described as well. Congenital heart disease is found in about 25% of cases. Mild dysmorphism such as arched eyebrows, broad nasal bridge, upturned nares, and prominent forehead are found in some patients. In addition macrocephaly and cleft lip and/or palate have been reported.

The core duplicated interval in the syndrome comprises 26 HUGO Gene Nomenclature Committee (HGNC) and four micro RNA (MIR) genes. Of the HGNC genes four appear to be dosage sensitive, i.e. GATA-binding protein 4 (*GATA4*, OMIM*600576), Tankyrase, TRF1-Interacting, Ankyrin-Related ADP-Ribose Polymerase (*TNKS1*, OMIM*60330), SRY-box 7 transcription factor (*SOX7*, OMIM*612202), and XK, Kell Blood Group Complex Subunit-Related Family, Member 6 (*XKR6*). Increased expression of these genes is thought to account for features observed in the 8p23.1 duplication syndrome [1]. *TNKS1* is implicated in behavioral anomalies [2], *SOX7* in developmental delay, and *GATA4* in concert with *SOX7* might cause congenital heart disease [2,3]. The role, if any, of *XKR6* is not known in the 8p23.1 duplication syndrome. Furthermore, MIR genes *MIR124-1* and *MIR598* might contribute to compromised neurocognition in the syndrome. However, nothing is known about their expression level. The other two MIR genes (*MIR597, MIR1322*) within this region are not known to be involved in developmental processes that are disturbed in the 8p23.1 duplication syndrome.

Here we describe a patient with a 1.80 Mbp *de novo* duplication within 8p23.1 and typical features of the 8p23.1 duplication syndrome. The duplicated region includes *TNKS1* and *SOX7* but not *GATA4* and only a portion of *XKR6*.

## Case presentation

The propositus was born at 41 weeks of gestation. His body weight was 2800 g (100 g below 3^rd^ centile), length 48 cm (3^rd^ percentile) and head circumference 35.5 cm (25^th^ to 50^th^ percentile). He is the third child of non-consanguineous healthy parents. A breech necessitated delivery by caesarean section. His older siblings, a brother and a sister, are healthy.

Muscular hypotonia was noticed during infancy. Motor development was delayed. He started to crawl late and began to walk during the third year of life. Speech development was also delayed. He started to vocalize simple words such as “mama” and “papa” well after three years of age. A recently performed hearing test was normal. At age 7 his developmental state was assessed according to the Denver Developmental Screening Test. Fine motor skills and speech corresponded to a 6 year old. Social competence and gross motor development were delayed by 1.5 years.

**Figure 1:**
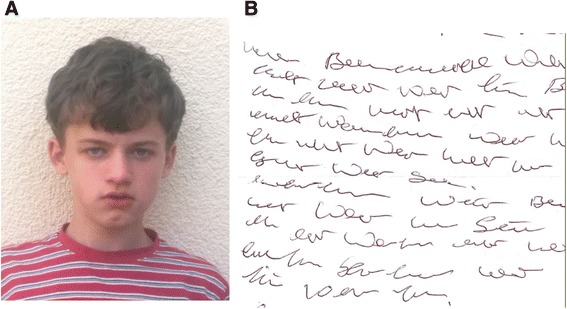
**Clinical findings. A**: Patient’s head, note broad nasal ridge, prominent forehead and dimple at chin. **B**: Nonsensical text produced by patient during office visit.

He is currently 11 years old and attends fourth grade in a school for children with special needs. His concentration span is short and memory retention is reduced as compared to healthy children of the same age. He displays bursts of aggressive behavior usually directed towards his sister. Otherwise he barely interacts with his siblings and classmates. He is able to read and understand simple sentences. While in the office with his mother he concentrated on writing and produced a nonsensical text (Figure 1B). During the last few years his motor skills have improved markedly. He is now able to swim and ride a bicycle. He is interested in machines and dexterous at performing manual tasks. His appearance is mostly normal. He has a broad nose, a prominent forehead, and a dimple on his chin (Figure 1A).

Informed consent was given by the patient’s mother to report this case.

## Results

**Figure 2:**
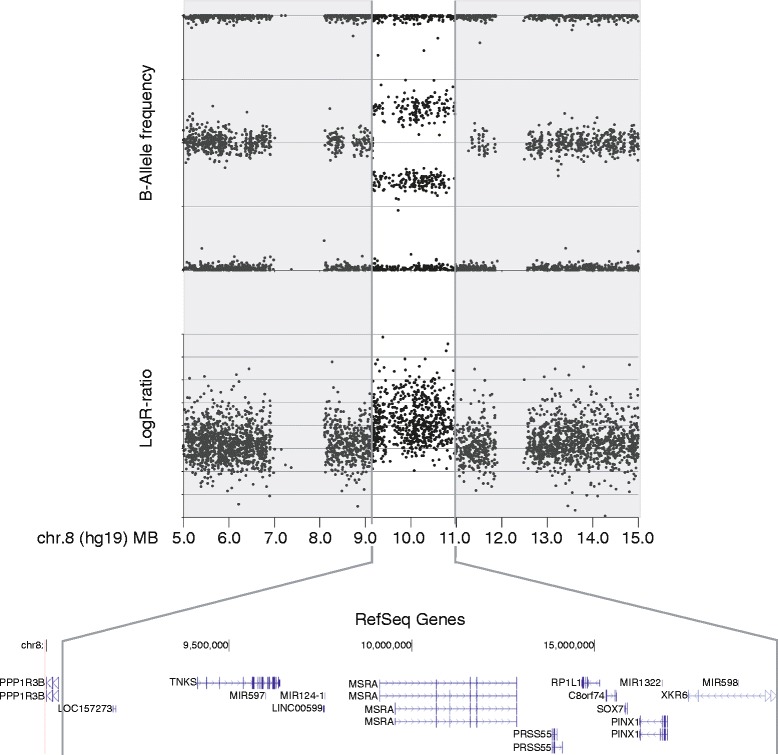
**Genomic region of the duplication in 8p23.1 identified by SNP-array.** Included are the dosage sensitive genes *TNKS1*, *SOX7*, and *XKR6* and four micro RNA genes *(MIR597*, *MIR124*, *MIR1322*, and *MIR598)*.

SNP array analysis revealed a 1.80 Mbp duplication in 8p23.1 spanning the region (hg19) from 9,169,154 to 10,969,075 (Figure 2) (arr[hg19]8p23.1 (9,169,154-10,969,075x3)dn). The region is covered by 1070 SNPs in total and comprises one truncated and 13 complete genes. Included in the duplication are *TNKS1*, *SOX7*, and truncated *XKR6* and the four micro RNA genes *MIR597*, *MIR124*, *MIR1322*, and *MIR598*. The duplication breakpoint lies within intron 1 of *XKR6* between SNP rs7460507 (10,969,075) and SNP rs2409709 (10,979,553). These SNPs are 10.4 kb apart. The distal breakpoint is defined by SNP rs3102070 (9,167,175) and SNP rs922269 (9,169,154) which are separated by 1.9 kb of DNA. The duplication has occurred de novo. The SNP arrays of the patient’s parents were normal.

45 out of 1070 SNPs within the duplicated region could be used for analysis of the parental origin of the duplication (criterion 1: mother and father homozygous for allele A = AA or B = BB; criterion 2: child heterozygous AAb or aBB) (Additional file 1: Table S1). All SNPs analyzed were of maternal origin. Thus, the duplication occurred on a maternal chromosome 8.

## Discussion

The patient has typical features of the 8p23.1 duplication syndrome. Both motor and linguistic development are markedly delayed. This is found in >90% of patients. He is intellectually disabled. In addition he displays symptoms of autism spectrum disorder which has not been reported in other cases [1,4,5]. He is not able to control his emotions and barely interacts with other children. His self-absorbed writing of a nonsensical text during the office visit further supports the diagnosis autism spectrum disorder (Figure 1B).

The duplicated region in the patient (1.80 Mbp) is about half the size of the previously defined critical interval (3.68 Mbp) delineated in currently 12 thoroughly investigated patients [summarized in 1]. DECIPHER Database currently lists 31 cases with 8p23.1 duplications overlapping the region found in the patient. The newly defined interval includes 7 HGNC and 4 MIR genes. Of the dose sensitive genes it contains *SOX7* and *TNKS1* but not *GATA4. MIR124-1* and *MIR598* that potentially contribute to disease are also within this interval. *XKR6* is only partially included since the proximal duplication breakpoint lies within the gene. The patient carries one truncated (only exons 2 to 3) and two complete copies of this gene. *XKR6* is therefore unlikely to contribute to the phenotype.

It is noteworthy that the duplication in 8p23.1 duplication syndrome is of maternal origin both in the present patient and in two previously studied cases [1].

Given that the patient displays typical features of the 8p23.1 duplication syndrome, the two genes *TNKS1* and *SOX7* might suffice to cause motor and linguistic developmental delay, mild intellectual disability, and minor dysmorphic features. A contributing role of *MIR124-1* and *MIR598* is possible*.* Both genes have been implicated in neuropsychiatric disorders and might be a contributing factor to autism spectrum disorder in the patient. However, nothing is known about their function when present in three copies. Consistent with normal expression of *GATA4* that functions in cardiac development the patient does not have congenital heart disease. Other signs and symptoms observed in some cases of the 8p23.1 duplication syndrome are also missing in the present case. These include ADHD, ocular anomalies, cleft lip and palate and seizures.

## Conclusion

In conclusion the finding of a duplication within 8p23.1 half the size of the common duplication in the 8p23.1 duplication syndrome further documents an important pathogenic role of *SOX7* and *TNKS1*. Absence of congenital heart disease is consistent with two copies and thus normal expression of *GATA4* in the patient.

## Methods

Genome-wide single nucleotide polymorphism (SNP) array analysis was performed using the HumanOmniExpress Bead Chip array (Illumina, Inc., San Diego, CA) according to manufacturer’s instructions. In brief, 200 ng of genomic DNA were amplified, fractionated, hybridized, and fluorescence-tagged. After scanning of the slides further analysis was carried out using the software (GenomeStudio and KaryoStudio) provided by Illumina. Genotype and copy number were calculated by determination of B-allele frequency and log_2_ - R ratio.

## Consent

Written informed consent was obtained from the patient’s parents for publication of this report and the accompanying images. A copy of the written consent is available for review by the Editor-in-Chief of this journal.

## Additional file

Additional file 1: Table S1. Origin of alleles in the duplicated region of 8p23.1.

## Abbreviations

pCNV: Pathogenic copy number variant
ADHD: Attention deficit hyperactivity disorder
HGNC: HUGO Gene Nomenclature Committee
MIR: Micro RNA
OMIM: Online Mendelian Inheritance in Man
SNP: Single nucleotide polymorphism
DECIPHER: Database of Chromosomal Imbalance and Phenotype in Humans using Ensembl Resources
