# Supplementary material for: 8p23.1 duplication syndrome: narrowing of critical interval to 1.80 Mbp
Source: Mol Cytogenet. 2014 Dec 9;7:94. doi: 10.1186/s13039-014-0094-3 (PMC4268894; doi:10.1186/s13039-014-0094-3)
Supplement: Additional file 1: Table S1. — Origin of alleles in the duplicated region of 8p23.1. [file 13039_2014_94_MOESM1_ESM.pdf]

Supplemental table 1: Origin of alleles in the duplicated region of 8p23.1

SNP	Chr	SNP position	allele propositus	allele mother	allele father	duplicated allele origin
rs12543276	8	9178921	AAb	AA	BB	maternal
rs11778929	8	9184529	aBB	BB	AA	maternal
rs10106829	8	9184628	aBB	BB	AA	maternal
rs6999153	8	9193501	AAb	AA	BB	maternal
rs12235038	8	9237403	AAb	AA	BB	maternal
rs4240625	8	9242557	aBB	BB	AA	maternal
rs17730481	8	9276163	aBB	BB	AA	maternal
rs10105543	8	9323515	aBB	BB	AA	maternal
rs7007562	8	9328926	AAb	AA	BB	maternal
rs7819054	8	9336714	AAb	AA	BB	maternal
rs7830939	8	9380132	aBB	BB	AA	maternal
rs9644677	8	9411808	AAb	AA	BB	maternal
rs9329203	8	9412153	AAb	AA	BB	maternal
rs6990097	8	9412857	AAb	AA	BB	maternal
rs10110146	8	9685362	AAb	AA	BB	maternal
rs4841239	8	9692825	aBB	BB	AA	maternal
rs7015101	8	9718278	AAb	AA	BB	maternal
rs661310	8	9787808	AAb	AA	BB	maternal
rs615171	8	9796189	aBB	BB	AA	maternal
rs13273152	8	9831907	aBB	BB	AA	maternal
rs7813389	8	9834862	AAb	AA	BB	maternal
rs17737355	8	9838936	AAb	AA	BB	maternal
rs537231	8	9839731	aBB	BB	AA	maternal
rs7815168	8	9847606	aBB	BB	AA	maternal
rs4517127	8	9862430	aBB	BB	AA	maternal
rs6987670	8	9883177	aBB	BB	AA	maternal
rs11787370	8	10030902	aBB	BB	AA	maternal
rs11993663	8	10032894	aBB	BB	AA	maternal
rs1905630	8	10033264	aBB	BB	AA	maternal
rs7820236	8	10044801	AAb	AA	BB	maternal
rs11249973	8	10046638	AAb	AA	BB	maternal
rs17748464	8	10050618	aBB	BB	AA	maternal
rs6982806	8	10056664	AAb	AA	BB	maternal
rs10097315	8	10060638	AAb	AA	BB	maternal
rs7832976	8	10065961	AAb	AA	BB	maternal
rs4380891	8	10338924	aBB	BB	AA	maternal
rs11250018	8	10340111	AAb	AA	BB	maternal
rs6601469	8	10343863	aBB	BB	AA	maternal
rs13282174	8	10345753	aBB	BB	AA	maternal
rs4392860	8	10356449	aBB	BB	AA	maternal
rs10086002	8	10391917	aBB	BB	AA	maternal
rs7011457	8	10407928	AAb	AA	BB	maternal
rs4841380	8	10418039	aBB	BB	AA	maternal
rs9329228	8	10482372	AAb	AA	BB	maternal
rs7386213	8	10503525	AAb	AA	BB	maternal
